# Acute Effect of a Dietary Multi-Ingredient Nootropic as a Cognitive Enhancer in Young Healthy Adults: A Randomized, Triple-Blinded, Placebo-Controlled, Crossover Trial

**DOI:** 10.3389/fnut.2022.858910

**Published:** 2022-05-12

**Authors:** María Medrano, Cristina Molina-Hidalgo, Juan M. A. Alcantara, Jonatan R. Ruiz, Lucas Jurado-Fasoli

**Affiliations:** ^1^PROmoting FITness and Health Through Physical Activity Research Group, Department of Physical Education and Sports, Faculty of Sport Sciences, Sport and Health University Research Institute (iMUDS), University of Granada, Granada, Spain; ^2^Department of Physiology, Faculty of Medicine, University of Granada, Granada, Spain; ^3^Instituto de Investigación Biosanitaria, ibs.Granada, Granada, Spain

**Keywords:** cognitive performance, ergogenic aid, dietary supplement, psychomotor performance, emotions, stress

## Abstract

**Aim:**

To study the acute effect of a dietary multi-ingredient nootropic on cognitive performance in young healthy adults. We also analyzed the influence of the dietary multi-ingredient nootropic on emotional state, heart rate (HR), and heart rate variability (HRV).

**Methods:**

This is a randomized, triple-blinded, placebo-controlled, crossover trial. In total, 26 young healthy adults (50% women; 24.9 ± 3.3 years old) ingested 10 g of a dietary multi-ingredient nootropic [Evo-Gamers^®^; Harrison Sport Nutrition (HSN), Granada, Spain] or placebo (maltodextrin) in a randomized order (clinicaltrials.gov No. NCT04790188). After 30 min of the ingestion, participants performed a battery of cognitive performance tests to measure the processing speed, inhibitory control, working memory, cognitive flexibility, creativity, and verbal fluency. The emotional status was assessed through questionnaires, and HR and HRV were measured using a heart rate monitor.

**Results:**

In comparison with placebo, the acute ingestion of the nootropic showed a significantly better response time in several cognitive tests (i.e., processing speed, inhibitory control, spatial working memory, and cognitive flexibility, all *P* < 0.05 and effect size range of 0.4–0.6). It also displayed a higher accuracy in the processing speed, the inhibitory control, and cognitive flexibility tests (all *P* < 0.05; effect size ranged from 0.4 to 0.6). Furthermore, the nootropic showed a higher creativity and positive emotions and lower sadness-depression emotions, whereas HR and HRV remained similar between placebo vs. nootropic conditions. However, there were no differences between the nootropic and placebo in verbal fluency, motivation, or anxiety (all *P* > 0.05).

**Conclusion:**

An acute ingestion of a dietary multi-ingredient nootropic enhances cognitive performance in comparison with placebo without negatively influencing HR or HRV in young healthy adults.

## Introduction

Recent evidence indicates that cognitive enhancers, such as nootropics, are increasingly used to improve or maintain brain health (e.g., cognitive performance, psychosocial and emotional status, etc.) (1, 2). Cognition refers to the capacity for information processing, applying knowledge, and changing preferences (2). Cognitive performance is composed of the intermingling of different processes such as memory, attention, executive functions, creativity, and intelligence cognitive functions (3). This cognitive performance is dependent on an interplay between the genotype and lifestyle factors, including cognitive activity, physical activity, sleep habits, and diet (4, 5). The primary use of nootropics has been focused on the treatment of cognitive deficits, commonly associated with neurodegenerative disorders or aging (1, 2). However, nootropics are being currently used among healthy individuals and athletes with the aim to enhance their performance. In fact, nootropics (or “smart drugs”) can improve memory, attention, creativity, or motivation through their action on neurotransmitters (i.e., dopaminergic, glutamatergic/cholinergic, and serotonergic systems), hormones, transduction systems, and brain metabolism (1, 3).

Although pharmacological nootropics have been demonstrated to enhance cognition, their chronic use may be limited due to the relatively high cost and their associated side effects (1). To solve these problems, food-based or dietary nootropics have emerged as alternative cognitive enhancer substances (1). Some dietary nootropics such as L-theanine (6), tyrosine (7), Huperzine A (8), taurine (9), and caffeine (10) have been proved to enhance cognitive functions in different populations. Nevertheless, these dietary nootropics have been investigated in isolation, but studies investigating the effects of a multi-ingredient dietary nootropic on cognitive performance in healthy individuals are scarce.

In modern society, the rise of the new generation of professionals with high-cognitive demand tasks and the high information from the media and internet have increased the demand for nootropics to help people to sustain cognitive performance (3). Achieving optimal performance during cognitively demanding tasks could be of great interest, especially for population groups whose occupations depend on cognitive performance such as students, university staff, or eSports gamers among others. We hypothesized that, based on previous studies and the composition of the supplement, the acute ingestion of a dietary multi-ingredient nootropic could enhance cognitive performance in healthy young adults. Thus, the main aim of this study was to examine the acute effect of a dietary multi-ingredient nootropic on different cognitive performance processes including processing speed, inhibitory control, spatial working memory, cognitive flexibility, creativity, working memory, and verbal fluency in young healthy adults. As a secondary outcome, we analyzed the influence of the dietary multi-ingredient nootropic on emotional state and on heart rate (HR) and heart rate variability (HRV).

## Materials and Methods

### Participants

A total of 26 young healthy adults (aged 20–35 years) from Granada (Spain) were recruited through social media advertisements. Potential participants were contacted using a designated email and asked to fill out preliminary details allowing us to predict the compatibility of the participants to the experiment. All participants were without current or past medical, neurological, and psychiatric disorders in their personal and family history. All denied use of prescription medications or illicit substances. Also, participants should meet the following criteria to be included: (i) having a body mass index from 18.5 to 30 kg/m^2^; (ii) being between 18 and 35 years old; (iii) having a stable body weight during the last 5 months (body weight changes <3 kg); (iv) being free of chronic diseases (i.e., diabetes, hypertension, etc.); (v) not being pregnant or lactating; (vi) not receiving pharmacological treatment with antihypertensive, lipid-lowering, hypouricemic, antidiabetic, beta-blockers, or neurological drugs; (vii) not being allergic or intolerant to any ingredient of the nootropic supplement, and (viii) not having any uncontrolled condition that, under the criterion of the researcher, prevents an adequate collection of the objective data of the study of this project. All inclusion and exclusion criteria were asked in a personal and familiar medical history and were self-reported by the participant. Habitual consumption of caffeinated drinks was allowed although standardized prior testing. Moreover, participants should be able to speak and read Spanish fluently.

This study was registered in clinicaltrials.gov (No. NCT04790188), and it was conducted in accordance with the last Declaration of Helsinki (11). Written informed consent was obtained from all participants after a full explanation of study procedures, possible adverse events, legal rights and responsibilities, and their right to voluntary termination. The University of Granada Research Ethics Committee approved this project (No. 2026/CEIH/2021).

### Study Design

**FIGURE 1:**
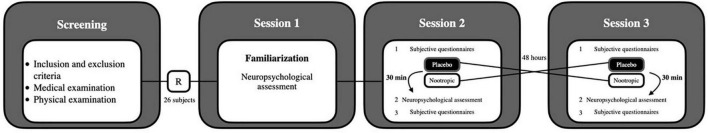
Study design. After screening, 26 subjects were randomized (R) in a triple-blind, cross-over trial.

This study had a randomized, triple-blinded (i.e., participants, research staff, and statistic staff), placebo-controlled, crossover design, involving three different sessions (Figure 1). The placebo and nootropic were coded and blinded by an external investigator, with no participation in this study, previously to be given. The study took place at the Sport and Health University Research Institute, iMUDS (Granada, Spain), from April 2021 to June 2021. Each session was scheduled during the morning at the same hour.

Initially, previous to the start of the main study, a test-retest experiment was performed to determine the possible learning effect in the computerized cognitive tasks using a separate sample of 8 healthy subjects (who met the abovementioned inclusion criteria). Participants of the test-retest experiment visited the research center 2 days with 48 h difference between days. During each visit, participants performed all the computerized cognitive tasks without the ingestion of any substance and strictly following the previously mentioned instructions. Since we observed a moderate learning effect in some outcomes (Supplementary Table 1), we decided to include a familiarization cognitive session to decrease the influence of this effect on the nootropic results (i.e., main study).

Therefore, after the initial screening, each participant of the main study completed a familiarization cognitive session (*Session 1*) and two sessions (*Sessions 2* and *3*) separated by 48 h. During the familiarization cognitive session (*Session 1*), all participants performed the computerized cognitive test without ingesting the nootropic or placebo. In this *Session 1*, participants’ weight and height were recorded using a Seca model 799 electronic column scale and stadiometer (Seca, Hamburg, Germany), and their body mass index was calculated. Subjects were asked to be barefoot and to wear only light clothing during these measurements. Then, dual-energy X-ray absorptiometry (Hologic Discovery Wii, Hologic, Bedford, MA, United States) was used to determine participants’ lean (kg) and fat mass (kg). All subjects also completed the international physical activity questionnaire (12).

During *Sessions 2* and *3*, the participants randomly ingested either 10 g of the dietary multi-ingredient nootropic Evo-Gamers [Harrison Sport Nutrition (HSN) Store, Granada, Spain; refer to Supplementary Table 2 for the detailed description of the supplement] or 10 g of placebo (Maltodextrin, HSN; same flavor and visual appearance than the nootropic beverage). The main ingredients of the nootropic were L-tyrosine, acetyl L-carnitine, HCL, citicoline sodium, alpha-glycerylphosphorylcholine (GPC), taurine, caffeine, L-theanine, extract from mango leaves, and extract from huperzia leaves (Supplementary Table 2). Of note, all participants ingested both the dietary multi-ingredient nootropic and the placebo. Participants completed two conditions in a counterbalanced order at approximately the same time of the day. Both, the nootropic and placebo were dissolved in 400 ml of water and served in the same recipients. The order of administration of the nootropic or placebo was randomized using a function in MS Excel for Windows^®^. Moreover, during these sessions, participants completed a number of subjective emotional and mood state questionnaires before and after the nootropic/placebo ingestion. The start of the cognitive testing was 30 min after the ingestion of the nootropic or the placebo [T_*max*_ of caffeine range: 30–120 min (13)]. All participants were contacted by personal message (i.e., mobile or email) 24 h after the session to assess late-occurring side effects, such as sleep disturbances, diarrhea, or stomachache.

All participants were provided with the following instructions prior to all sessions: (i) to have breakfast at least 2 h prior to the assessments and to replicate the breakfast during all cognitive sessions; (ii) to avoid stimulants 12 h prior to the cognitive assessments; (iii) to avoid vigorous physical activity the 24 h before the test days; (iv) to avoid the consumption of alcohol or drugs 24 h prior to the assessments; and (v) to try to sleep at their “habitual” sleep hours. Compliance with these instructions was checked by self-reported dietary and exercise records before all the study sessions.

### Cognitive Performance Assessments

The cognitive performance tests were conducted according to fixed processing times for each test and without breaks in between. The order of the tests in each session performed was randomized using a function in MS Excel for Windows^®^ to minimize possible cognitive fatigue effects (14, 15).

#### Processing Speed

To measure the processing speed, a simple reaction time and choice reaction time (determination test) tasks were evaluated using the Vienna Test System (Schuhfried GmbH, Mödling, Austria). In the simple response time (RT) condition, a manual response (to press a yellow circle button) was required to react to visual circle stimuli. In the determination test, participants were required to respond to rapidly changing visual and acoustic stimuli. Each stimulus was presented for three fixed periods of time, namely, (i) 1,078 ms in Interval 1 (RTI1), (ii) 834 ms in RTI2, and (iii) 948 ms in RTI3, and a total of 120 stimuli were presented for each condition. Manual response (circles buttons) was required to react to visual circle stimuli, also manual response (rectangular buttons) was required to two different acoustic stimuli, and finally, the pedal response was required to react to visual rectangular stimuli.

#### Inhibitory Control

To measure inhibitory control, a Stroop test was conducted (16) using the E-Prime^®^ software^1^. Participants watched single words appear on the screen written in either red, blue, or green ink. After every word appeared, the participant was asked to indicate whether the color of the word was either red, blue, or green. There were three conditions, namely, congruent (written word = ink color), incongruent (written word ≠ ink color), and neutral (the word was not a color altogether). This test evaluated executive control, specifically attention and inhibitory control.

#### Working Memory

To evaluate the cognitive process of working memory, we used N-Back and spatial working memory tests in the abovementioned E-Prime software. In the spatial working memory test, a group of 2, 3, or 4 black dots flashed on the screen. Then, one red dot flashed on the screen. In each attempt, the participant was asked to indicate whether the red dot flashes were located in the place where a black dot had also just flashed (17). In the N-Back test, a serial presentation of a stimulus (i.e., an alphabetic letter) was spaced several seconds apart. The participant had to decide whether the current stimulus matched the one displayed “*n*” trials ago, where *n* was a variable number that could be adjusted up or down to, respectively, increase or decrease the cognitive load. We used 1-back and 2-back in this study (18).

#### Cognitive Flexibility

Cognitive flexibility was tested using two different computerized tests, namely, Task switch and Flankers in the E-Prime^®^ software (16, 19) and also using the Trail Making Test (TMT) in paper (20, 21). The Task switch test asked participants to learn two different tasks. In the first task, a number was presented in a circle, and the participant had to indicate whether the number was lower or higher than 5. For the second task, a number was presented in a square, and the participant had to indicate whether the number is odd or even. After practicing these two tasks, the participant completed a block of circle-only trials, then a block of square-only trials, and finally the circle and square trials were intermixed (16). In the Flankers test, stimuli were assigned to one of two responses, and the participant was required to respond to the target stimulus when this was flanked by other stimuli (19). The stimuli were presented at a known location (usually at fixation), and the flanking stimuli were associated with a response that was either the same as or different from that assigned to the target.

The Trail Making Test was composed of two parts, namely, (i) the TMT A, where the targets were all numbers from 1 to 25 and the participant had to connect them in a sequential order, and (ii) TMT B, where the dots went from 1 to 13 and include letters from A to L. As in the first part, the participant had to connect the dots in order while alternating letters and numbers, e.g., 1-A-2-B-3-C, in the shortest time possible without lifting the pen from the paper.

#### Creativity

Creativity and divergent thinking were measured with the Creativity IQ-Test (CREA) test (22, 23). The CREA is theoretically based on the classic factors of creativity (i.e., divergent production, flexibility, fluency, and originality) and in approaches to problem formulation, lateral thinking, and study of cognitive styles. According to the authors of this test, the creative psychological style supposes a general disposition of the subject for the openness and versatility of cognitive schemes. To evaluate this, CREA asks people to formulate the as many questions as possible about a stimulus visual. Each question supposes a new cognitive scheme emerging from the interaction between the stimulus and ability of the subject to open that information to which he/she already has. The CREA proposes a unitary measure of creativity, aimed at evaluating the openness and versatility of schemes cognitions that emerge during the task of formulating questions. The outcome measure in this task was the total number of questions reported.

#### Verbal Fluency

To measure verbal fluency, we used a short test of verbal functioning (24). It consisted of two tasks, namely, letter fluency (sometimes called *phonological fluency*) and category fluency (*semantic fluency*). In the standard versions of the tasks, participants had 1 min to produce as many unique words as possible starting with a given letter (*phonological fluency*) or within a semantic category (*semantic fluency*). The participant’s score in each task was the number of unique correct words. For this study, we used the phonological fluency: /f/, /a/, /s/, /m/, /r/, /p/ and the semantic verbal fluency test: /animals/ and /fruits/.

### Emotional State Assessment

The emotional state was evaluated through different questionnaires: (i) *subjective affect* was evaluated with items from the Positive and Negative Affect Schedule (PANAS) (25, 26); (ii) *motivation* was measured with the Situational Motivation Scale (EMSI) (27); (iii) *mood state* was evaluated with the Spanish version of mood state (28); and (iv) *anxiety* was measured by State-Trait Anxiety Inventory (STAI) (29).

### Heart Rate and Heart Rate Variability Assessment

Before the ingestion of the nootropic or placebo, the heart rhythm (i.e., HR and HRV) was measured in resting conditions for a 15 min period with a Polar RS800CX HR monitor (Polar Electro Oy, Kempele, Finland) in a quiet room. A sampling frequency of 1,000 Hz was used, and the R-R intervals series were detrended using the smoothness prior method with alpha set at 500. Before and during the assessment, participants were instructed to breathe normally and not to talk, fidget, or sleep while measurements were being taken. Later, the heart rhythm was continuously monitored during the whole sessions (i.e., before and after the beverage intake).

To derive HR and HRV from the recorded heart rhythm, we used the Kubios software (v.3.0.0, HRV analysis, University of Eastern Finland) (30, 31) and the medium threshold-based artifact correction (i.e., medium Kubios filter), as has it been proposed for young adults (32). For the baseline heart rhythm recording (i.e., before the beverage intake), the best 5 min period of was manually selected by one trained evaluator (JMAA) based on the following criteria: (i) Gaussians R-R intervals and HR distribution graphs; (ii) no large R-R interval outliers; and (iii) R-R intervals equidistance (33). Then, the heart rhythm recorded during the cognitive tests (i.e., after the beverage intake) was manually selected by the same evaluator (JMAA). The central period of each measurement during the cognitive test was selected [i.e., simple reaction time (4 min), determination test (9 min), Stroop test (7 min), N-Back test (7 min), spatial working memory test (12 min), Flankers test (4 min), Task switch test (10 min), TMT (4 min), verbal fluency (10 min), and CREA (5 min)].

Using the aforementioned Kubios software (30, 31), in the time domain, we computed: (i) the standard deviation of all normal R-R intervals (SDNN) in milliseconds (ms) and (ii) the squared root of the mean of the sum of the squares of successive normal R–R interval differences (RMSSD) in ms. Finally, we also derived the HR in beats per minute (bpm).

### Statistical Analysis

Sample size and power calculations were determined based on the results of prior studies (34–36). A medium effect size of 0.62 was estimated. Therefore, to achieve the statistical power and to investigate differences between the acute ingestion of the nootropic or the placebo, a minimum of 22 participants were required (power = 95% and α = 0.05) (G-Power 3.1.5 software). To achieve the statistical power taking into account possible dropouts of some participants, a total of 26 young adults were recruited (50% women).

Descriptive characteristics of the participants were presented as means and standard deviations (for continuous variables) or frequencies and percentages (for categorical variables). We studied whether significant sex interactions were presented in the cognitive outcomes. Since no sex interactions were observed (all *P* > 0.05), data of men and women were analyzed together. Due to the non-normal nature in the distribution of the variables (Shapiro–Wilk test, all *P* < 0.05), the comparison in the outcomes between the ingestion of the nootropic or the placebo was performed using the Wilcoxon test. A delta (Δ) was computed as Δ_*n–p*_ = nootropic – placebo, and the effect size of the Wilcoxon test was calculated as *r* = z/√N (37). All the analyses were performed using the Statistical Package for Social Sciences (SPSS, version 23.0; SPSS Inc., Chicago, IL, United States), and the level of significance was set at *P* < 0.05.

## Results

Table 1 shows the descriptive characteristics of the participants.

**TABLE 1:** Descriptive characteristics of the study participants (*N* = 26).

Age	24.9 (3.3)
Women (*n*, %)	13, 50
Dietary supplements ingestion (*n*, %)	9, 36
Caffeine (*n*)	1
Creatine (*n*)	2
Whey protein (*n*)	3
Pea protein (*n*)	1
Vitamin D (*n*)	1
Vitamin B12 and omega-3 (*n*)	1
Sociodemographic characteristics	
Smoker (*n*, %)	6, 23
Physical activity (METS/day)	3,570 (2,719)
Occupation (*n*, %)	
Student	19, 73
Researcher	4, 16
Teacher	1, 4
Computer programmer	2, 8
Anthropometric characteristics	
Height (cm)	167.8 (10.1)
Weight (kg)	65.3 (11.9)
Body mass index	22.8 (2.5)
Lean mass (kg)	43.8 (11.5)
Fat mass (%)	29.3 (8.8)

*Data are expressed as mean (standard deviation) or frequencies (percentages).*

*METS, metabolic equivalent of task derived from the International Physical Activity Questionnaire.*

### Effect of the Dietary Multi-Ingredient Nootropic on Cognitive Performance

**FIGURE 2:**
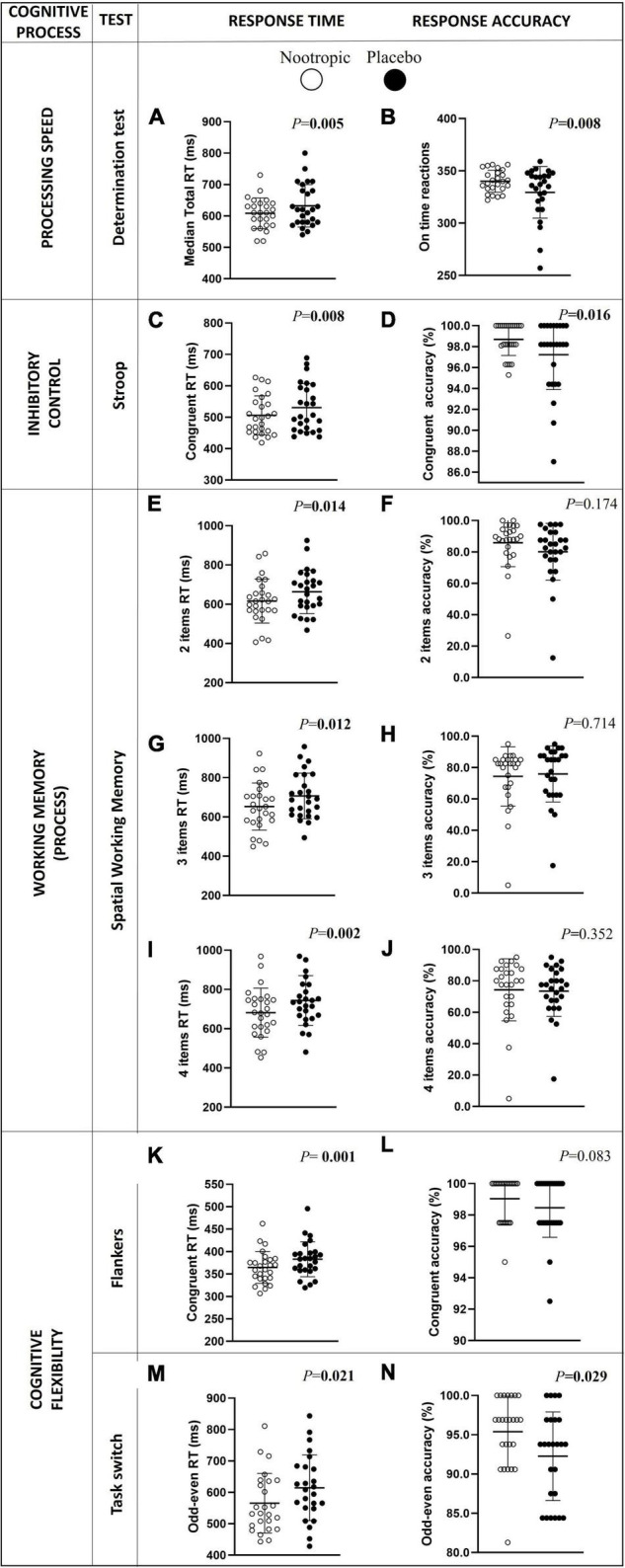
Differences in choice processing speed (A,B), inhibitory control (C,D), spatial working memory (D–J), and cognitive flexibility RT (K–N) and accuracy between the acute ingestion of the nootropic (denoted as white circles) and the placebo (denoted as black circles). Circles represent individual values, whereas lines represent mean (i.e., central line) and standard deviation. Differences between nootropic and placebo were analyzed using the Wilcoxon test. Boldfaced values mean *P* < 0.05. RT, response time.

After the acute ingestion of the nootropic, and in comparison with the placebo, participants showed a lower RT in the following cognitive processes: processing speed measured by the determination test (Δ_*n–p*_ = -24.4 ± 37.9 ms; *r* = 0.545; *P* < 0.01; Figure 2A), inhibitory control (Δ_*n–p*_ = -24.4 ± 51.9 ms; *r* = 0.520; *P* = 0.008 for congruent condition, Figure 2C; and Δ_*n–p*_ = -17.7 ± 42.2 ms; *r* = 0.436; *P* = 0.026 for neutral condition, Supplementary Table 3), spatial working memory (Δ_*n–p*_ = -47.2 ± 107.2 ms and *r* = 0.481 for item 2; Δ_*n–p*_ = -53.9 ± 102.4 and *r* = 0.491 for item 3; Δ_*n–p*_ = -61.9 ± 105.5 ms and *r* = 0.615 for item 4; all *P* < 0.014, Figures 2E,G,I), and cognitive flexibility (Δ_*n–p*_ = -18.3 ± 22.0 ms and *r* = 0.650 in Flankers test; Δ_*n–p*_ = -51.6 ± 93.2 ms and *r* = 0.431 in the odd-even part of the Task switch test; all *P* < 0.03, Figures 2K,M, respectively).

Additionally, participants showed a higher response accuracy in the processing speed (Δ_*n–p*_ = 11 ± 21 reactions on time; *r* = 0.518; *P* < 0.01, Figure 2B), the inhibitory control (Δ_*n–p*_ = 1.5 ± 3.0 congruent answers; *r* = 0.471; *P* < 0.01, Figure 2D), and cognitive flexibility (odd-even Task switch) (Δ_*n–p*_ = 3.4 ± 6.3 congruent answers; *r* = 0.353; *P* < 0.03, Figure 2N) after the acute ingestion of the nootropic in comparison with placebo. However, the response accuracy in tests of the spatial working memory or cognitive flexibility (Flankers and Task switch tests) was similar after the ingestion of the nootropic and the placebo (all *P* > 0.05, Figures 2F,H,J,L, respectively).

The positive effects after the acute ingestion of the nootropic were also displayed on a lower RT and higher reactions on-time of all intervals of the determination test (all *r* > 0.4 and *P* < 0.05; Supplementary Table 3) in comparison with the placebo ingestion. Furthermore, the RT was lower after the ingestion of the nootropic in comparison with the placebo in the inhibitory control (i.e., Stroop test) and the cognitive flexibility (i.e., Flankers and Task switch tests) (all *r* > 0.4 and *P* < 0.03; Supplementary Table 3). However, no differences were observed after the acute ingestion of the nootropic or the placebo in working memory (i.e., N-Back test) and verbal fluency tests (all *P* > 0.05; Supplementary Table 3).

**FIGURE 3:**
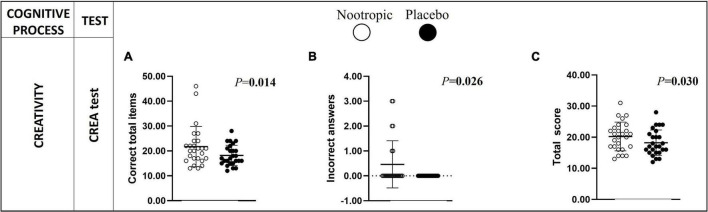
Differences in creativity between the acute ingestion of the nootropic (denoted as white circles) and the placebo (denoted as black circles). Creativity was assessed with the CREA test involving the correct total items (A), incorrect answers (B), and the total score (C). Circles represent individual values, whereas lines represent mean (i.e., central line) and standard deviation. Differences between nootropic and placebo were analyzed using the Wilcoxon test. Boldfaced values mean *P* < 0.05. CREA, Creativity IQ-Test.

In terms of creativity, after the acute ingestion of the nootropic, participants displayed a significantly higher number of correct total responses (Δ_*n–p*_ = 4 ± 7 correct words; *r* = 0.483; *P* < 0.02, Figure 3A), incorrect answers (Figure 3B) and a higher total score (Δ_*n–p*_ = 2 ± 4 score; *r* = 0.425; *P* < 0.03, Figure 3C) in comparison with the placebo.

### Effects of the Dietary Multi-Ingredient Nootropic on Emotional Status

**FIGURE 4:**
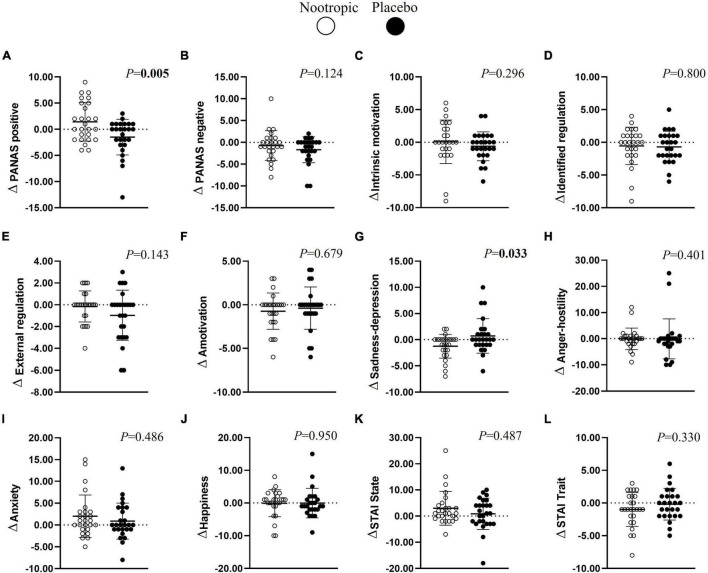
Effects of a nootropic ergogenic aid (denoted as white circles) in comparison with a placebo (denoted as black circles) on emotional status differences (Δ = after beverage − before beverage) between before and after the performance of the cognitive test by the participants. Emotional status include positive (A) and negative (B) emotions, intrinsic motivation (C), identified (D) and external regulation (E), amotivation (F), sadness-depression (G), anger-hostility (H), anxiety (I), happiness (J), anxiety state (K), and anxiety trati (L). Circles represent individual values, whereas lines represent mean (i.e., central line) and standard deviation. Differences between nootropic and placebo were analyzed using the Wilcoxon test. Boldfaced values mean *P* < 0.05. PANAS, Positive and Negative Affect Schedule; STAI, State-Trait Anxiety Inventory.

After the cognitive tests, the acute ingestion of the nootropic increased positive emotions (Δ_*n–p*_ = 2.9 ± 1.0; *r* = 0.530; *P* < 0.01, Figure 4A) and decreased the emotions related to sadness-depression (Δ_*n–p*_ = -2.0 ± 0.8; *r* = 0.414; *P* < 0.04, Figure 4G) in comparison with the placebo. However, the nootropic did not influence negative emotions, motivation, anger-hostility, happiness, or anxiety emotions (all *P* > 0.05, Figure 4).

### Effects of the Dietary Multi-Ingredient Nootropic on Heart Rate and Heart Rate Variability

The acute ingestion of the nootropic did not influence the HR during the completion of the cognitive tests except for the HR during the completion of the working memory N-back test (Δ_*n–p*_ = -3 ± 4 bpm; *r* = 0.138; *P* < 0.01, Supplementary Table 4) and the RT test (Δ_*n–p*_ = -3 ± 8 bpm; *r* = 0.148; *P* < 0.001, Supplementary Table 4). Similarly, there were no differences in the HRV (RMSSD and SDNN) after the nootropic or the placebo, except for the RMSSD during the completion of the working memory N-back test (Δ_*n–p*_ = 9 ± 18 ms; *r* = 0.110; *P* < 0.04, Supplementary Table 4).

Finally, Supplementary Table 5 shows the prevalence of side effects after the acute ingestion of the nootropic and the placebo.

## Discussion

This study shows that the studied dietary multi-ingredient nootropic (1) improves the RT in different cognitive tests; (2) improves accuracy in processing speed, inhibitory control, and cognitive flexibility tests; (3) improves creativity; (4) increases positive emotions and decreases sadness-depression emotions; and (5) does not affect HR in young healthy adults. In comparison with placebo, the dietary multi-ingredient nootropic does not influence verbal fluency, motivation, or anxiety in young healthy adults. Since the effect size of the nootropic was medium (*r* range 0.4-0.6), these results suggest that this dietary multi-ingredient nootropic could be used as a cognitive enhancer in situations where an increase in cognitive performance is needed (i.e., students, university staff, eSports gamers, etc.).

Previous studies have shown that supplementation with caffeine (38), the combination of caffeine and taurine (34), caffeine and L-theanine (39), tyrosine (40), and mango leaves extract (41) can improve the RT in different cognitive and computerized tests. In this study, we showed that a dietary multi-ingredient nootropic improves the processing speed of information in young healthy adults. This effect could be due to the synergic effects of different dietary ingredients included in the nootropic (34, 38–41). Also, it could be partially explained by the central nervous system activation and alertness enhancement effect of caffeine, which could increase the processing speed and decrease the RT (38). It is important to highlight that this potential activation of the central nervous system was not accompanied by an increase in the HR or decrease in the HRV (as a proxy of physiological stress) in our study participants. Indeed, this could be explained by the inclusion of L-theanine as a dietary ingredient in the nootropic because it can counteract the potential effects derived from a high dose of caffeine on anxiety (42), psychological stress (43), blood pressure (42), and HR (43), which completely agrees with our results.

Our study also shows that the dietary multi-ingredient nootropic improved the accuracy, specifically in the determination test, in the inhibitory control test, in the spatial working memory test, and in the cognitive flexibility tests. In this sense, the accuracy improvement observed in the determination test was observed in interval 2, which is an “under pressure” situation, due to the short period of time between stimuli, and in interval 3 which is the last part of the test and evaluates the maintained attention (44). This improvement could be partially explained by the synergic effects of the individual dietary components, which have been demonstrated to improve accuracy in different cognitive tests mainly due to their improvement in attention and concentration [i.e., citicoline and alpha-GPC (45, 46) mango leaves extract (41), L-tyrosine (7, 47), and the combination of taurine and caffeine (9)]. However, a recent meta-analysis has demonstrated that caffeine decreases the RT without improving the response accuracy of cognitive tests measuring attention, simple reaction time, and inhibitory control in athletic populations (10). This does not concur with our results since we observed positive effects of the nootropic on the accuracy of the determination test, inhibitory control, and cognitive flexibility. Additionally, we demonstrated that this dietary multi-ingredient nootropic does not worsen the response accuracy in different cognitive tests in comparison with placebo, even improving the response accuracy in some cognitive tests (i.e., incongruent condition of the Stroop test).

We also observed that the dietary multi-ingredient nootropic simultaneously increases creativity and positive emotions and decreases sadness-depression emotions. In previous studies, both caffeine and tea (i.e., L-theanine) increased creativity in young students (48–50). In contrast, evidence about different dietary compounds on emotions is mixed. In this sense, caffeine alone or combined with taurine did not modify the mood profile in some studies (51, 52), whereas it induced positive effects on mood profile in other studies (i.e., decreasing sadness and increasing positive emotions) (48, 53). Tea consumption has also been demonstrated to increase positive emotions in young students (49, 50). Taken all together, these results might partially concur with our findings. Unfortunately, no study has investigated the role of other dietary ingredients on creativity or emotions (i.e., citicoline, alpha-GPC, mango leaves extract, or Huperzia leaves extract), thus precluding further comparisons.

Interestingly, there is scarce evidence investigating the effects of multi-ingredients nootropics on cognitive performance. Most studies have investigated the role of individual dietary nootropics without the combination of different ingredients. In this sense, a previous study showed that a multi-ingredient choline-based nootropic (with huperzia extract, alpha-GPC, ginseng, and black pepper extract) delayed fatigue during a strength exercise (54). Furthermore, a stimulant-free multi-ingredient nootropic (with L-tyrosine, *Ginkgo biloba*, alpha-GPC, L-theanine, N-acetyl-L-carnitine HCL, theobromine, and picamilon) was as effective as caffeine supplementation for increasing cognitive function (i.e., alertness, focus, cognition, and memory) in healthy young males (55). Our results partially agree with these two studies, suggesting that the combination of different nootropic ingredients could be effective as a cognitive enhancer (i.e., memory, cognition, focus, positive emotions, and cognitive precision) in healthy populations.

### Strength and Limitations

The strength of this study is the inclusion of a familiarization day, which ensures that the effects of the dietary multi-ingredient nootropic are due to its effects and are not influenced by a learning effect. Additional strengths are the triple-blinding experimental design and the inclusion of wide-known cognitive tests. However, the study population included young healthy adults, which does not allow for potential extrapolation of the findings to older, high-level athletes, or unhealthy populations. Furthermore, the study design has its limitation due to the lack of individual comparator groups including in an isolated way all the dietary ingredients contained in the multi-ingredient nootropic.

### Practical Applications

In line with our results, the use of the dietary multi-ingredient nootropic could have different practical applications in situations where a cognitive enhancement is necessary. This dietary supplement could be used in situations where a quick response is needed, specifically where the processing speed of information is essential. In addition, it could be also used in different scenarios where high creativity is needed to solve problems, as well as situations where is critical to react as quickly as possible to different stimuli (i.e., videogames or sports performance). Therefore, this dietary multi-ingredient nootropic might be a useful tool in different sports practices, specifically eSports, where a quick and creative response is required. It could be also a potential dietary supplement used in highly demanding jobs with immediate response and with a short time for its execution (i.e., pilots, military, and computer programmers among others). However, further studies are needed to better understand if the dietary multi-ingredient nootropic could improve cognitive performance in specific tasks for these activities/occupations, as well as, in different populations. Future research lines could be: (i) to investigate the role of this dietary multi-ingredient nootropic in the improvement of cognitive performance in sports practices (i.e., eSports), military populations, or computer programmers and (ii) to evaluate the role of the nootropic as a cognitive enhancer in the context of different neurodegenerative diseases.

## Conclusion

This study shows that an acute ingestion of dietary multi-ingredient nootropic improves cognitive performance in young healthy adults. This dietary supplement improves the processing speed of information in different cognitive tests (i.e., inhibitory control and cognitive flexibility), increases the accuracy (i.e., inhibitory control and cognitive flexibility), increases creativity, and improves emotional status without affecting HR. Our results suggest that this supplement could be used as a cognitive enhancer in different situations where an increase in cognitive performance is needed.

## Data Availability Statement

The raw data supporting the conclusions of this article will be made available by the authors, without undue reservation.

## Ethics Statement

The study was reviewed and approved by the University of Granada Research Ethics Committee (No. 2026/CEIH/2021). The patients/participants provided their written informed consent to participate in this study.

## Author Contributions

LJ-F, CM-H, and JR conceived and designed the study. LJ-F, MM, and JA acquired the data. MM and JA elaborated the statically section. LJ-F, MM, and CM-H drafted the manuscript. All authors revised the manuscript, read, and approved the final manuscript.

## Conflict of Interest

The authors declare that the research was conducted in the absence of any commercial or financial relationships that could be construed as a potential conflict of interest.

## Publisher’s Note

All claims expressed in this article are solely those of the authors and do not necessarily represent those of their affiliated organizations, or those of the publisher, the editors and the reviewers. Any product that may be evaluated in this article, or claim that may be made by its manufacturer, is not guaranteed or endorsed by the publisher.

## Footnotes

1
https://www.pstnet.com/eprime.cfm
