# Supplementary material for: Acute Effect of a Dietary Multi-Ingredient Nootropic as a Cognitive Enhancer in Young Healthy Adults: A Randomized, Triple-Blinded, Placebo-Controlled, Crossover Trial
Source: Front Nutr. 2022 May 12;9:858910. doi: 10.3389/fnut.2022.858910 (PMC9133906; doi:10.3389/fnut.2022.858910)
Supplement: Supplementary file 1 [file Data_Sheet_1.docx]

Supplementary Material

**Table S1.** Learning effect analysis of processing speed, inhibitory control, working memory, cognitive flexibility, and verbal fluency in a control sample (n=8).

| **Cognitive process** | **Test** | | **Outcome** | **Test** | **Re-Test** | **Δ (Re-test – Test)** | **P** |
| --- | --- | --- | --- | --- | --- | --- | --- |
| Processing speed | Simple reaction time | Median RT (ms) | | 295.3 (23.0) | 288.3 (23.2) | -7.0 (25.8) | 0.263 |
|  |  | Correct (%) | | 97.8 (2.7) | 100.0 (0.0) | 2.2 (2.7) | 0.059 |
|  |  | Incorrect (%) | | 3.6 (5.1) | 0.0 (0.0) | -3.6 (5.1) | 0.109 |
|  | Determination test | Total - Median RT (ms) | | 670.0 (44.4) | 631.3 (29.0) | -38.7 (23.0) | **0.011** |
|  |  | Total On time reactions (%) | | 77.5 (32.3) | 99.3 (0.7) | 21.7 (33.1) | **0.011** |
|  |  | Int 1 - Median RT (ms) | | 673.8 (50.4) | 632.5 (24.4) | -41.2 (37.6) | **0.027** |
|  |  | Int 1 - On time reactions (%) | | 97.2 (0.2) | 99.3 (0.7) | 2.1 (1.3) | 0.115 |
|  |  | Int 2 - Median RT (ms) | | 671.3 (33.6) | 623.8 (35.1) | -48.0 (22.5) | **0.011** |
|  |  | Int 2 - On time reactions (%) | | 85.2 (10.7) | 94.1 (5.3) | 8.9 (2.3) | **0.012** |
|  |  | Int 3 - Median RT (ms) | | 671.3 (51.1) | 627.5 (30.6) | -43.7 (26.2) | **0.011** |
|  |  | Int 3 - On time reactions (%) | | 93.3 (9.1) | 95.8 (3.0) | 2.5 (2.6) | 0.495 |
| Inhibitory control | Stroop | Congruent RT (ms) | | 649.2 (91.8) | 606.5 (96.6) | -42.7 (71.3) | 0.161 |
|  |  | Congruent accuracy (%) | | 98.8 (1.4) | 97.0 (4.5) | -1.9 (4.0) | 0.216 |
|  |  | Incongruent RT (ms) | | 732.7 (103.7) | 680.1 (128.2) | -52.6 (81.8) | 0.123 |
|  |  | Incongruent accuracy (%) | | 92.2 (5.8) | 94.00 (6.7) | 1.8 (8.9) | 0.672 |
|  |  | Neutral RT (ms) | | 655.9 (95.8) | 634.6 (94.9) | -21.3 (84.4) | 0.889 |
|  |  | Neutral Accuracy (%) | | 97.6 (2.8) | 96.4 (4.8) | -1.2 (2.8) | 0.263 |
| Working memory | N-Back | 1-back RT (ms) | | 898.4 (289.6) | 763.87 (84.4) | -134.6 (230.6) | 0.093 |
|  |  | 1-back accuracy (%) | | 65.0 (18.5) | 72.08 (18.5) | 7.1 (21.8) | 0.445 |
|  |  | 2-back RT (ms) | | 1087.2 (241.1) | 1084.6 (289.5) | -2.7 (258.7) | 0.889 |
|  |  | 2-back accuracy (%) | | 65.2 (28.4) | 81.7 (19.3) | 16.5 (20.3) | **0.026** |
|  | SWM | 2-item RT (ms) | | 760.5 (200.2) | 688.1 (119.4) | -72.0 (102.8) | 0.093 |
|  |  | 2-item accuracy (%) | | 80.3 (12.1) | 80.0 (21.5) | -0.3 (16.3) | 0.050 |
|  |  | 3-item RT (ms) | | 767.6 (164.1) | 691.3 (94.5) | -76.3 (86.0) | 0.208 |
|  |  | 3-item accuracy (%) | | 79.7 (8.3) | 73.8 (25.1) | -5.9 (21.6) | 0.865 |
|  |  | 4-item RT (ms) | | 815.0 (158.5) | 773.2 (117.4) | -41.8 (80.7) | 0.599 |
|  |  | 4-item accuracy (%) | | 71.6 (11.3) | 71.6 (25.0) | 0.0 (21.0) | 0.612 |
| Cognitive Flexibility | Flankers | Congruent RT (ms) | | 418.9 (55.5) | 409.8 (48.6) | -9.2 (22.2) | 0.401 |
|  |  | Congruent accuracy (%) | | 99.1 (1.9) | 99.1 (1.9) | 0.0 (1.3) | 1.000 |
|  |  | Incongruent RT (ms) | | 488.1 (49.7) | 464.7 (42.9) | -23.4 (25.5) | **0.036** |
|  |  | Incongruent accuracy (%) | | 95.7 (6.5) | 93.8 (6.0) | -0.9 (4.2) | 0.546 |
|  |  | Neutral RT (ms) | | 421.9 (49.9) | 409.2 (42.6) | -12.8 (25.7) | 0.327 |
|  |  | Neutral Accuracy (%) | | 98.1 (2.6) | 98.4 (1.9) | 0.3 (2.5) | 0.705 |
|  | Task switch | Low-high RT (ms) | | 625.9 (112.2) | 554.0 (54.0) | -72.0 (85.1) | 0.050 |
|  |  | Low-high accuracy (%) | | 98.8 (1.6) | 98.44 (2.4) | -0.4 (3.1) | 0.705 |
|  |  | Odd-even RT (ms) | | 659.0 (58.7) | 648.0 (74.1) | -2.0 (78.7) | 0.889 |
|  |  | Odd-even accuracy (%) | | 98.4 (2.4) | 95.7 (3.3) | -2.7 (3.5) | 0.066 |
|  |  | Repetition RT (ms) | | 890.4 (66.1) | 832.7 (78.8) | -57.7 (54.2) | **0.036** |
|  |  | Repetition accuracy (%) | | 92.2 (5.2) | 93.6 (4.3) | 1.4 (5.3) | 0.600 |
|  |  | Switch RT (ms) | | 1015.4 (72.3) | 872.6 (79.1) | -142.7 (77.6) | **0.012** |
|  |  | Switch accuracy (%) | | 89.8 (9.7) | 92.6 (5.1) | 2.8 (10.7) | 0.398 |

Differences between between the test and re-test were analyzed using the test of Wilcoxon. Boldfaced values: P < 0.05. *Abbreviations*: RT, response time; ms, milliseconds; Int 1, interval 1; Int 2, interval 2; Int 3, interval 3; SWM, Spatial Working Memory test.

| **Table S2***.* Nutritional composition of the Evo-Gamers^®^ nootropic | |
| --- | --- |
| L-tyrosine | 1000 mg |
| Acetyl L-Carnitine HCL | 500 mg |
| Citicoline sodium | 200 mg |
| L-alpha glycerylphosphorylcholine (Alpha-GPC) | 100 mg |
| Vitamin C | 810μg |
| Vitamin E | 12.1mg |
| Vitamin B6 | 3 mg |
| Vitamin B2 | 3 mg |
| Vitamin A | 810μg |
| Vitamin D | 10μg |
| Vitamin B12 | 5μg |
| Taurine | 500 mg |
| Caffeine | 300 mg |
| L-Theanine | 150 mg |
| Mango Leaves (*Mangifera indica*) | 2000 mg |
| Extract 20:1 from Mangifera indica leaves (60% Mangiferin) | 100mg |
| Mangiferin | 60mg |
| Huperzia Leaves (*Huperzia serrata*) | 200 mg |
| Extract 40:1 from Huperzia serrata leaves (1% Huperzin A) | 5mg |
| Huperzin A | 50μg |

**Table S3.** Effect of a dietary multi-ingredient nootropic on variables of processing speed, inhibitory control, working memory, cognitive flexibility, and verbal fluency.

| **Cognitive process** | **Test** | **Outcome** | **Nootropic** | **Placebo** | **Δ_n-t_** | **Effect**  **size (r)** | **P** |
| --- | --- | --- | --- | --- | --- | --- | --- |
| Processing speed | Simple reaction time | Median RT (ms) | 264.5 (39.5) | 277.8 (56.6) | -13.8 (42.) | 0.207 | 0.290 |
|  |  | Correct answers | 27.9 (0.3) | 27.7 (0.7) | 0.2 (0.7) | 0.277 | 0.157 |
|  |  | Incorrect answers | 0.0 (0.0) | 0.0 (0.0) | 0.0 (0.0) | 0.000 | 1.000 |
|  | Determination test | Median RT (interval 1) (ms) | 612.8 (52.9) | 630.000 (68.2) | -18.0 (40.3) | 0.404 | **0.040** |
|  |  | On time reactions (interval 1) | 116.4 (3.0) | 115.9 (3.6) | 0.6 (3.5) | 0.193 | 0.326 |
|  |  | Median RT (interval 2) (ms) | 606.8 (47.9) | 630.8 (68.5) | -24.0 (39.3) | 0.495 | **0.012** |
|  |  | On time reactions (interval 2) | 109.8 (5.9) | 103.8 (14.748) | 6.2 (12.0) | 0.496 | **0.011** |
|  |  | Median RT (interval 3) (ms) | 606.8 (50.4) | 635.0 (71.5) | -29.2 (46.5) | 0.531 | **0.007** |
|  |  | On time reactions (interval 3) | 113.960 (4.5) | 109.7 (8.0) | 4.40 (7.2) | 0.539 | **0.006** |
| Inhibitory control | Stroop | Incongruent answers RT (ms) | 598.2 (129.9) | 623.0 (135.4) | -24.8 (68.9) | 0.286 | 0.144 |
|  |  | Incongruent answers accuracy | 1.0 (0.0) | 0.9 (0.1) | 0.0 (0.1) | 0.171 | 0.382 |
|  |  | Neutral RT (ms) | 538.9 (70.5) | 556.6 (85.2) | -17.7 (42.2) | 0.436 | **0.026** |
|  |  | Neutral accuracy | 1.0 (0.0) | 1.0 (0.0) | 0.0 (0.0) | 0.249 | 0.204 |
|  |  | Inhibitory control RT (ms) | -92.2 (83.1) | -92.6 (79.3) | 0.3 (51.7) | 0.032 | 0.869 |
|  |  | Inhibitory control accuracy | 0.0 (0.0) | 0.0 (0.1) | 0.0 (0.1) | 0.135 | 0.492 |
| Working memory | N-Back | RT (1 back item) (ms) | 743.8 (179.6) | 790.4(224.3) | -47.0 (243.8) | 0.119 | 0.545 |
|  |  | Accuracy (1 back item) | 0.7 (0.2) | 0.7 (0.2) | 0.0 (0.2) | 0.156 | 0.426 |
|  |  | RT (2 back items) (ms) | 939.1 (209.9) | 1014.0 (272.2) | -74.8 (199.6) | 0.281 | 0.151 |
|  |  | Accuracy (2 back items) | 85.2 (20.1) | 82.4 (20.7) | 2.77 (13.6) | 0.182 | 0.353 |
| Cognitive Flexibility | Flankers | Incongruent answers RT (ms) | 426.4 (39.3) | 447.3 (47.0) | -20.9 (23.0) | 0.720 | **<0.001** |
|  |  | Incongruent answers accuracy | 0.9 (0.1) | 0.9 (0.1) | 0.004 (0.071) | 0.013 | 0.948 |
|  |  | Neutral RT (ms) | 370.2 (34.6) | 384.1 (36.1) | -13.9 (21.2) | 0.605 | **0.002** |
|  |  | Neutral Accuracy | 1.0 (0.0) | 1.0 (0.0) | 0.0 (0.0) | 0.169 | 0.388 |
|  | Task switch | Low-high RT (ms) | 509.9 (108.1) | 547.7 (109.8) | -32.9 (72.7) | 0.377 | 0.054 |
|  |  | Low-high accuracy (%) | 1.0 (0.1) | 96.4 (4.5) | -1.0 (10.6) | 0.129 | 0.510 |
|  |  | Odd-even RT (ms) | 565.5 (94.8) | 614.6 (104.2) | -51.6 (93.2) | 0.454 | **0.021** |
|  |  | Odd-even accuracy (%) | 95.4 (4.5) | 92.3 (5.6) | 3.4 (6.3) | 0.429 | **0.029** |
|  |  | Repetition RT (ms) | 751.6 (118.8) | 786.5 (119.0) | -30.2 (110.4) | 0.269 | 0.170 |
|  |  | Repetition accuracy (%) | 0.832 (0.311) | 89.6 (15.1) | 3.1 (25.6) | 0.102 | 0.602 |
|  | TMT | Errors (part A) | 1 (2) | 1 (2) | 0 (2) | 0.341 | 0.069 |
|  |  | Errors (part B) | 3 (6) | 3 (4) | 0 (3) | 0.125 | 0.528 |
|  |  | Errors (B *minus* A) | 2 (5) | 2 (5) | 0 (3) | 0.081 | 0.678 |
| Verbal fluency | Verbal fluency | Correct answers (Phon.) | 91 (20) | 88 (21) | 3 (19) | 0.207 | 0.292 |
|  |  | Correct answers (Sem.) | 43 (7) | 42 (6) | 1 (5) | 0.225 | 0.252 |
|  |  | Total correct answers | 134 (25) | 130 (24) | 4 (21) | 0.239 | 0.223 |
|  |  | Incorrect answers (Phon.) | 4 (3) | 3 (3) | 1 (2) | 0.224 | 0.254 |
|  |  | Incorrect answers (Sem.) | 3 (2) | 2 (2) | 1 (2) | 0.316 | 0.107 |
|  |  | Total incorrect answers | 7 (5) | 5 (34) | 2 (3) | 0.390 | **0.047** |

Differences between nootropic and placebo were analyzed using the test of Wilcoxon. The effect size was calculated as r=z/√N. Boldfaced values: P < 0.05. Δ_n-t_ *=* nootropic – placebo*. Abbreviations*: RT, response time; TMT, Trail Making Test; Phon., phonologic; Sem., semantic.

**Table S4.** Effect of a nootropic ergogenic aid on heart rate (HR) and heart rate variability (HRV) outcomes in each of the performed cognitive tests.

| **Cognitive process** | **Test** |  | **HR and HRV outcomes** | **Nootropic** | **Placebo** | **Δ_n-t_** | **Effect**  **size (r)** | **P** |
| --- | --- | --- | --- | --- | --- | --- | --- | --- |
| Basal values | |  | Mean HR (bpm) | 73 (9) | 73 (11) | 0 (5) | 0.036 | 0.494 |
|  |  |  | RMSSD (ms) | 40 (18) | 40 (15) | 0 (14) | 0.002 | 0.968 |
|  |  |  | SDNN (ms) | 47 (18) | 44 (16) | 3 (10) | 0.085 | 0.107 |
| Processing  speed | Simple reaction time |  | Mean HR (bpm) | 67 (13) | 70 (10) | -3 (8) | 0.148 | **0.005** |
|  |  |  | RMSSD (ms) | 56 (25) | 51 (16) | 5 (20) | 0.064 | 0.227 |
|  |  |  | SDNN (ms) | 59 (21) | 56 (16) | 3 (17) | 0.020 | 0.376 |
|  | Determination test |  | Mean HR (bpm) | 70 (12) | 70 (11) | 0 (7) | 0.042 | 0.421 |
|  |  |  | RMSSD (ms) | 47 (16) | 45 (17) | 2 (17) | 0.049 | 0.355 |
|  |  |  | SDNN (ms) | 47 (13) | 48 (16) | -1 (15) | 0.015 | 0.778 |
| Inhibitory control | Stroop |  | Mean HR (bpm) | 67 (10) | 70 (10) | -3 (7) | 0.089 | 0.091 |
|  |  |  | RMSSD (ms) | 53 (22) | 50 (20) | 3 (15) | 0.053 | 0.314 |
|  |  |  | SDNN (ms) | 54 (16) | 55 (16) | 1 (15) | 0.021 | 0.968 |
| Working memory | N-back |  | Mean HR (bpm) | 67 (9) | 70 (11) | -3 (4) | 0.138 | **0.009** |
|  |  |  | RMSSD (ms) | 57 (19) | 48 (25) | 9 (18) | 0.110 | **0.036** |
|  |  |  | SDNN (ms) | 60 (17) | 54 (23) | 6 (18) | 0.083 | 0.117 |
|  | Spatial working memory |  | Mean HR (bpm) | 70 (11) | 70 (11) | 0 (5) | 0.068 | 0.199 |
|  |  |  | RMSSD (ms) | 51 (20) | 49 (25) | 2 (13) | 0.053 | 0.286 |
|  |  |  | SDNN (ms) | 53 (16) | 56 (20) | -3 (14) | 0.001 | 0.983 |
| Cognitive Flexibility | Flankers |  | Mean HR (bpm) | 66 (9) | 66 (10) | 0 (6) | 0.002 | 0.968 |
|  |  |  | RMSSD (ms) | 56 (21) | 51 (24) | 5 (13) | 0.076 | 0.147 |
|  |  |  | SDNN (ms) | 56 (16) | 54 (22) | 2 (17) | 0.013 | 0.809 |
|  | Task swtich |  | Mean HR (bpm) | 67 (9) | 69 (10) | -2 (6) | 0.061 | 0.243 |
|  |  |  | RMSSD (ms) | 54 (25) | 50 (22) | 4 (14) | 0.068 | 0.198 |
|  |  |  | SDNN (ms) | 55 (20) | 54 (17) | 1 (11) | 0.015 | 0.778 |
|  | TMT |  | Mean HR (bpm) | 70 (10) | 67 (10) | 3 (8) | 0.087 | 0.099 |
|  |  |  | RMSSD (ms) | 53 (20) | 48 (18) | 5 (25) | 0.053 | 0.314 |
|  |  |  | SDNN (ms) | 58 (17) | 52 (18) | 6 (22) | 0.076 | 0.147 |
| Verbal fluency | Verbal fluency |  | Mean HR (bpm) | 71 (11) | 69 (11) | 2 (7) | 0.074 | 0.159 |
|  |  |  | RMSSD (ms) | 48 (14) | 48 (15) | 0 (17) | 0.000 | 1.000 |
|  |  |  | SDNN (ms) | 54 (13) | 53 (15) | 1 (15) | 0.021 | 0.687 |
| Creativity | CREA |  | Mean HR (bpm) | 70 (10) | 68 (10) | 2 (8) | 0.057 | 0.277 |
|  |  |  | RMSSD (ms) | 51 (20) | 44 (14) | 7 (20) | 0.066 | 0.212 |
|  |  |  | SDNN (ms) | 55 (19) | 48 (15) | 7 (22) | 0.070 | 0.184 |

Differences between nootropic and placebo were analyzed using the test of Wilcoxon. Values are means (standard deviations) Boldfaced values: P < 0.05. Δ_n-t_ *=* nootropic – placebo. *Abbreviations*: HR, heart rate; HRV, heart rate variability; bpm, beats per minute; ms, milliseconds; RMSSD, Root mean square of successive RR interval differences; SDNN, Standard deviation of NN (intervals, inter-beat intervals from which artifacts have been removed) intervals; TMT, Trail Making Test.

**Table S5**. Side effects experienced by the participants of the study (N=26) after the acute ingestion of the nootropic and the placebo, and their prevalence comparison.

|  | **Nootropic** | |  | **Placebo** | |  | **P*** |
| --- | --- | --- | --- | --- | --- | --- | --- |
|  | N | % |  | N | % |  |  |
| Abdominal pain | 0 | 0 |  | 0 | 0 |  | - |
| Sickness | 0 | 0 |  | 0 | 0 |  | - |
| Vomiting | 0 | 0 |  | 0 | 0 |  | - |
| Dizziness | 0 | 0 |  | 0 | 0 |  | - |
| Itching | 0 | 0 |  | 0 | 0 |  | - |
| Rashes | 0 | 0 |  | 0 | 0 |  | - |
| Anxiety | 0 | 0 |  | 0 | 0 |  | - |
| Diarrhea | 6 | 23 |  | 0 | 0 |  | **0.009** |
| Headache | 0 | 0 |  | 1 | 4 |  | 0.313 |
| Chest pain | 2 | 8 |  | 0 | 0 |  | 0.149 |
| tachycardia | 0 | 0 |  | 0 | 0 |  | - |
| General malaise | 0 | 0 |  | 1 | 4 |  | 0.313 |
| Tremor | 1 | 4 |  | 0 | 0 |  | 0.313 |
| Numbness/tingling | 0 | 0 |  | 0 | 0 |  | - |
| Muscle cramps/aches | 0 | 0 |  | 0 | 0 |  | - |

*Differences in the prevalence of side effect between the acute intake of nootropic and placebo (chi square analysis).
